# Metagenomes of Microbial Communities in Arsenic- and Pathogen-Contaminated Well and Surface Water from Bangladesh

**DOI:** 10.1128/genomeA.01170-14

**Published:** 2014-11-20

**Authors:** Alice C. Layton, Archana Chauhan, Daniel E. Williams, Brian Mailloux, Peter S. K. Knappett, Andrew S. Ferguson, Larry D. McKay, M. Jahangir Alam, Kazi Matin Ahmed, Alexander van Geen, Gary S. Sayler

**Affiliations:** a Center for Environmental Biotechnology, University of Tennessee, Knoxville, Tennessee, USA; b Department of Microbiology, University of Tennessee, Knoxville, Tennessee, USA; c Department of Earth and Planetary Sciences, University of Tennessee, Knoxville, Tennessee, USA; d Joint Institute for Biological Sciences, University of Tennessee, Knoxville, and Oak Ridge National Laboratory, Knoxville, Tennessee, USA; e Department of Environmental Science, Barnard College, New York, New York, USA; f Department of Geology and Geophysics, Texas A&M University, College Station, Texas, USA; g Civil Engineering & Engineering Mechanics, Columbia University, New York, New York, USA; h Department of Geology, University of Dhaka, Dhaka, Bangladesh; i Lamont-Doherty Earth Observatory of Columbia University, Palisades, New York, USA

## Abstract

The contamination of drinking water from both arsenic and microbial pathogens occurs in Bangladesh. A general metagenomic survey of well water and surface water provided information on the types of pathogens present and may help elucidate arsenic metabolic pathways and potential assay targets for monitoring surface-to-ground water pathogen transport.

## GENOME ANNOUNCEMENT

Bangladesh has two microbial-based public health water resource crises. Diarrheal diseases (1), often attributable to poor sanitary conditions and fecal contamination of drinking water, remain a leading cause of mortality for children younger than 5 years (2, 3). In locations with poor sanitation, groundwater is a cleaner source than surface water; however, shallow wells may still contain waterborne pathogens (4). In parts of Bangladesh, the drinking water situation is further complicated by the presence of geogenic arsenic above drinking water standards (>10 µg/liter), which causes serious and debilitating diseases (5, 6). The goal of this study was to generate metagenomic sequences from arsenic- and pathogen-contaminated ground and surface waters in Bangladesh (7).

Surface and ground water samples were collected 4 times between March 2009 and November 2012 in a 1-km^2^ area of Bara Haldia, MatLab, Bangladesh (25.467°N 89.517°E) (4) representing both wet and dry seasons (8). In wells, the arsenic concentrations ranged from <1 to 218 parts per billion (ppb), and culturable *Escherichia coli* concentrations ranged from <1 to 100 most probable number (MPN)/100 ml. DNA was extracted from water filters using the Fast DNA spin kit (MP Biomedicals, CA) (2). DNA from 9 to 12 wells or 3 surface water samples was combined from each date to provide a composite sample. Metagenomic libraries were prepared using the Illumina Nextera DNA library preparation kit (Illumina, Inc., CA), and 2 × 100 bp reads were sequenced on an Illumina HiSeq 2000. The raw sequence reads from 8 metagenomic data sets were annotated using MG-RAST version 3.3.7 (9).

In surface water metagenomes, *Bacteria* was the dominant domain (94.2%), followed by *Eukaryota* (3.6%), DNA-based viruses (1.7%), and *Archaea* (0.4%). The most abundant genera in the surface water samples were *Acidovorax* (4.8%), *Mycobacterium* (4.1%), and *Burkholderia* (2.9%). Sequences matching well-known waterborne bacterial pathogens (10) found in surface water included: *Vibrio cholerae* and *Vibrio parahaemolyticus* (0.08%), *Salmonella enterica* (0.07%), *Clostridium difficile* (0.03%), *Cronobacter sakazakii* (0.03%), *Shigella flexneri* and *Shigella dysenteriae* (0.03%), *Staphylococcus aureus* (0.02%), *Campylobacter jejuni* (0.01%), and *Helicobacter pylori* (0.01%),

Consistent with the anaerobic conditions in ground water, archaea sequences comprised 20.4% of the sequences, with the genus *Methanosarcina* representing 4.1% of all well water metagenome sequences. Bacteria comprised 78.1%, eukaryotes 1.2%, and DNA-based viruses <0.1% of the sequences, with *Geobacter* (3.5%) and *Clostridium* (2.0%) being the second and third most abundant genera. Sequences matching the surface water pathogens were found in groundwater but generally at lower concentrations, which was expected due to die-off and filtering. Unexpectedly, four pathogens had 3- to 4-fold higher relative sequence abundances in ground than surface water: *C. difficile* (0.12%), *S. aureus* (0.04%) *C. jejuni* (0.03%), and *H. pylori* (0.03%).

In both surface and well water metagenomes, genes for arsenic metabolism, including arsenate reductase and arsenite oxidation, along with those for arsenic resistance, were present. These genetic pathways may affect arsenic mobility and toxicity (11). In summary, these metagenomic data sets have helped and can continue to further help elucidate the mechanisms of groundwater pathogen and arsenic contamination in Bangladesh.

### Nucleotide sequence accession number.

Nucleotide sequences obtained were deposited at the NCBI Sequence Read Archive under the accession no. SRP047074.
